# Downregulation of Immunosuppressive Environment in Patients with Chronic HBV Hepatitis on Maintained Remission

**DOI:** 10.3389/fimmu.2015.00052

**Published:** 2015-02-11

**Authors:** Belal Chaudhary, Eyad Elkord

**Affiliations:** ^1^United Arab Emirates University, Al Ain, United Arab Emirates; ^2^University of Salford, Manchester, UK; ^3^University of Cambridge, Cambridge, UK; ^4^University of Manchester, Manchester, UK

**Keywords:** immunosuppressive, Foxp3, PD1PD-L1, chronic HBV, downregulation

Recent years have witnessed the development of highly effective immunomodulatory therapies, especially for treating cancers and autoimmune diseases. The most promising targets include immunosuppressive Tregs, characterized by FoxP3 expression, and the inhibitory pathways involving CTLA-4 and PD-1/PD-L1 molecules. A recent paper by Germanidis et al. provides interesting insights into the relevance of these immunosuppressive pathways for treating chronic hepatitis B virus (HBV) hepatitis (CHB) (1).

CHB is characterized by chronic liver damage, inflammation, and fibrosis, eventually leading to cirrhosis and hepatic carcinoma. The HBV life cycle is not cytolytic to infected hepatocytes, and the liver damage is caused by a prolonged immune response induced by the expression of HBsAg on infected hepatocytes. The immune response involves HBsAg-specific CD8^+^ CTLs and to a lesser extent CD4^+^ T effector cells (Teff) (2). CHB is managed with either interferon-based therapies that act by enhancing anti-viral immune responses or nucleos(t)ide analogs (NAs), such as entecavir, which inhibit HBV replication (3). Successful anti-viral therapy is characterized by (i) restored anti-viral immune response, (ii) HBsAg seroconversion, (iii) a decrease in covalently closed circular DNA (cccDNA), and (iv) a decrease in circulating HBV DNA (3–5). However, most patients initially experience “remission,” followed by HBV reactivation after treatment withdrawal (3). cccDNA is the HBV transcription template, it is highly stable and persists even following resolution of CHB or acute HBV infection. It enables HBV reactivation and it is a key hurdle for achieving complete remission (3).

Elevated levels of intra-hepatic and circulating FoxP3^+^ Tregs have been described in CHB and chronic hepatitis C (CHC) (6–8). In this study, Germanidis et al. reported down-regulated liver mRNA expression of FoxP3 and suppressive cytokines, IL-10, and TGF-β, in patients maintained on-treatment following 5 years of remission compared to patients with active disease and no prior treatment (1). CD8 was also decreased in patients on-treatment and in remission. This data suggest a decrease in both intra-hepatic FoxP3^+^ Tregs and CTLs following CHB resolution. Interestingly, IL-2 and IFN-γ expressions were not restored during remission; this could indicate long-term CTL impairment or else it could be due to a reduction in intra-hepatic CTLs preventing any immune response from being restored to pre-infection intensity.

The role of Tregs in CHB and other chronic viral infections is not fully clear and further analyses are required (9, 10). *In vitro* Treg depletion can restore the functional activity of virus-specific CTLs (11, 12). In addition, Tregs are expanded in severe CHB, but correlate with serum viral load rather than impaired HBV-specific immune responses (6, 7). In this study, Germanidis et al. proposed that FoxP3^+^ Tregs might be expanded non-specifically in response to chronic liver inflammation rather than as HBV-specific Tregs (1). In a previous study, the same group reported that liver FoxP3 expression is closely linked to liver inflammation regardless of the underlying cause; viral, toxic, or autoimmunity (13). The current findings confirm FoxP3 is strongly correlated with inflammation in CHB, in addition to PD-1, PD-L1, and CD8. FoxP3 expression also correlated closely with serum viral load, ALT, and AST (markers of liver injury). Apoptosis-induced inflammation is observable in CHB and other liver diseases due to sustained liver injury (14), and Tregs could prevent catastrophic pathology due to apoptosis-induced inflammation. During inflammatory expansion, HBV-specific Tregs might also be generated in response to HBsAg on infected hepatocytes explaining differing reports regarding HBV-specific and non-specific Tregs from CHB patients.

FoxP3 is essential for Treg development and it is used as the hallmark marker to identify Tregs in most studies. However, FoxP3 can be upregulated on non-suppressive Teff during inflammation and activation. More specific Treg markers have been identified, as recently reviewed (15). Therefore, more robust phenotypic and functional identification of intra-hepatic suppressive Treg subsets is critical, and future studies should utilize robust markers, *in vitro* suppression assays or TSDR methylation to confirm the suppressive Treg status.

This study also reported a positive correlation of PD-1, PD-L1, and the apoptotic mediators FAS and FAS-L with inflammation intensity (1). The PD-1/PD-L1 pathway is prominent in immune tolerance by promoting Treg development and T cell dysfunction (16). PD-1 and PD-L1 are upregulated, respectively, on “exhausted” CTLs in various pathological settings and on virally infected hepatocytes (17, 18). PD-1 upregulation has been characterized during chronic liver inflammation and, similar to the proposed mechanism for Treg expansion, upregulation of the PD-1/PD-L1 pathway might represent a protective mechanism against chronic inflammation in CHB (17). Tumor-necrosis factor-related apoptosis-inducing ligand (TRAIL) was upregulated during remission and negatively correlated with inflammation. This might be representative of enhanced hepatocyte destruction during inflammation. Further investigation into TRAIL expression in CHB and chronic viral inflammation is needed. The immunosuppressive mechanisms in CHB active disease and their resolution upon remission are summarized in Figure 1.

**Figure 1:**
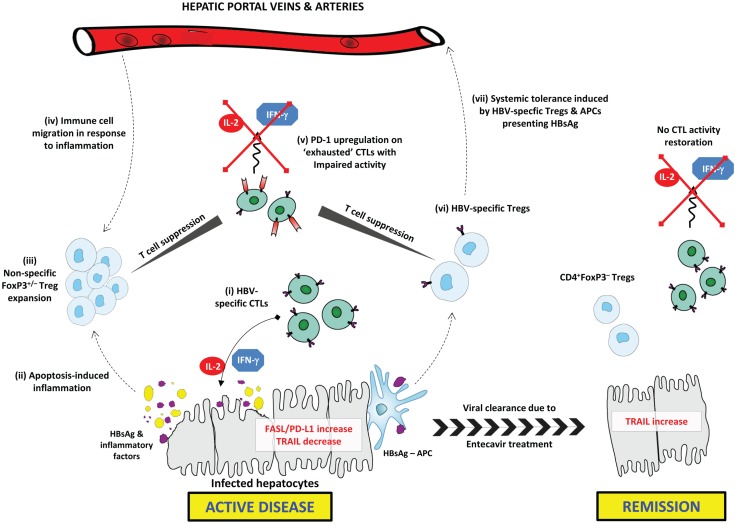
**Immunosuppressive mechanisms in CHB active disease and their resolution upon remission**. *In active CHB disease*, FAS-L and PD-L1 expression are increased on hepatocytes, while TRAIL is down-regulated. (i) HBsAg-specific CTLs secrete effector cytokines (IL-2 and IFN-γ) inducing apoptosis in infected hepatocytes expressing HBsAg. (ii) Excessive apoptosis and pro-inflammatory cytokine secretion induces chronic inflammation, and the release of inflammatory factors. HBsAg is released from lysed hepatocytes. (iii) Tregs are expanded in response to inflammatory factors; this may include both FoxP3^+/−^ Treg subsets. Tregs exert non-specific CTL suppression. (iv) Circulating Tregs and other immune cells, including NK cells and MDSCs, migrate toward the site of inflammation contributing to impaired HBV-specific immune responses. (v) CTLs exhibit an “exhausted” anergic phenotype characterized by upregulated PD-1 expression. Exhausted CTLs are unable to exert any immune activity including secretion of effector cytokines. (vi) HBsAg-presenting APCs induce generation of HBV-specific Tregs that selectively suppress HBV-specific CTLs. (vii) Circulating HBV-specific Tregs and HBsAg–APCs are able to induce systemic tolerance to HBsAg, thus further delaying HBV clearance. *Upon remission*, FAS-L and PD-L1 expression are down-regulated, while TRAIL is upregulated. CD4^+^FoxP3^–^ T cells may be expanded or persist following resolution of inflammation, while CTLs do not regain similar functional capacity to pre-infection.

It is important to take into account the significant heterogeneity of Treg subpopulations. Both FoxP3^+^ Tregs and FoxP3^−^ Tregs, such as Tr1 and Th3, exist. Tissue-specific hepatic Tregs have also been described (19). Germanidis et al. note that in patients in remission, there is no corresponding decrease in CD4 with FoxP3 as might be expected with CD4^+^FoxP3^+^ Treg reduction. Peripheral Tregs (pTregs), as opposed to thymic Tregs (tTregs), are known to be expanded during chronic inflammation (20). Tregs expanded due to chronic inflammation could be FoxP3^–^ pTregs in addition to FoxP3^+^ pTregs. Moreover, FoxP3 might also be reduced due to resolved inflammation.

The unique immune function of the liver should also be considered when investigating CHB immunobiology. Various subsets of immune cells continuously traffic into and out of the liver interacting with APCs, including Kupffer cells and LSECs. This complex network of APCs and immune-modulating cells maintain a tolerogenic environment and are able to induce systemic tolerance in response to intra-hepatic antigen presentation (21). Breaking liver-induced systemic HBV tolerance might be key to restoring effective anti-HBV immune responses.

The study by Germanidis et al. raises some interesting questions regarding immune regulation in CHB. What is the nature of TRAIL and the FAS/FAS-L pathway in HBV clearance? What is the role of other immune regulatory cells in CHB immune tolerance? Most importantly, are Tregs and immune-modulating pathways promoted as a result of chronic inflammation or the cause for delayed HBV clearance?

With regards to Tregs as a therapeutic target, further investigations are required. Tregs only comprise 1% intra-hepatic immune cells; expanded B regulatory cells, NK cells, and myeloid-derived suppressor cells have all been characterized in CHB and CHC (8). Immunomodulation could provide a novel therapeutic approach for CHB and chronic viral infections. Interestingly, antibody blockade of the PD-1/PD-L1 pathway restored the activity of intra-hepatic CTLs *in vitro* (18, 22). However, significant challenges remain in balancing immune activation to clear HBV infection without inducing further liver injury (23, 24).

## Conflict of Interest Statement

The authors declare that the research was conducted in the absence of any commercial or financial relationships that could be construed as a potential conflict of interest.
